# Taking action on violence through research, policy, and practice

**DOI:** 10.1186/s41256-016-0006-7

**Published:** 2016-07-15

**Authors:** Ilene Hyman, Mandana Vahabi, Annette Bailey, Sejal Patel, Sepali Guruge, Karline Wilson-Mitchell, Josephine Pui-Hing Wong

**Affiliations:** 1 Dalla Lana School of Public Health, University of Toronto, Toronto, Canada; 2 Daphne Cockwell School of Nursing, Toronto, Canada; 3 Centre for Global Health and Health Equity, Toronto, Canada; 4 Diverse and At Risk Population, Toronto, Canada; 5 Research Cluster, Ryerson University, Toronto, Canada; 6 Daphne Cockwell School of Nursing, Toronto, Canada; 7 Centre for Global Health and Health Equity, Ryerson University, Toronto, Canada; 8 Early Childhood Studies, Toronto, Canada; 9 Centre for Global Health and Health Equity, Ryerson University, Toronto, Canada; 10 Urban Health, Daphne Cockwell School of Nursing, Toronto, Canada; 11 Centre for Global Health and Health Equity, Toronto, Canada; 12 Nursing Centre for Research and Education on Violence Against Women and Children, Ryerson University, Toronto, Canada; 13 College of New Scholars, Artists and Scientists, Royal Society of Canada, Toronto, Canada; 14 Department of Health Sciences, The Open University of Sri Lanka, Toronto, Canada; 15 Midwifery Education Program, Toronto, Canada; 16 Centre for Global Health and Health Equity, Ryerson University, Toronto, Canada; 17 Daphne Cockwell School of Nursing, Ryerson University, Toronto, Canada; 18 Adjunct Professor, Dalla Lana School of Public Health, OHTN-CIHR New Investigator, Toronto, Canada; 19 Centre for Global Health and Health Equity, Ryerson University, Toronto, Canada

**Keywords:** Structural violence, Social policy, Immigration, Community services, Neoliberalism

## Abstract

**Background:**

Violence is a critical public health problem associated with compromised health and social suffering that are preventable. The Centre for Global Health and Health Equity organized a forum in 2014 to identify: (1) priority issues related to violence affecting different population groups in Canada, and (2) strategies to take action on priority issues to reduce violence-related health inequities in Canada. In this paper, we present findings from the roundtable discussions held at the Forum, offer insights on the socio-political implications of these findings, and provide recommendations for action to reduce violence through research, policy and practice.

**Methods:**

Over 60 academic researchers, health and social service agency staff, community advocates and graduate students attended the daylong Forum, which included presentations on structural violence, community violence, gender-based violence, and violence against marginalized groups. Detailed notes taken at the roundtables were analyzed by the first author using a thematic analysis technique.

**Findings:**

The thematic analysis identified four thematic areas: 1) structural violence perpetuates interpersonal violence - the historical, social, political and economic marginalization that contributes to personal and community violence. 2) social norms of gender-based violence—the role of dominant social norms in perpetuating the practice of violence, especially towards women, children and older adults; 3) violence prevention and mitigation programs—the need for policy and programming to address violence at the individual/interpersonal, community, and societal levels; and 4) research gaps—the need for comprehensive research evidence made up of systematic reviews, community-based intervention and evaluation of implementation research to identify effective programming to address violence.

**Conclusions:**

The proceedings from the Global Health and Health Equity Forum underscored the importance of recognizing violence as a public health issue that requires immediate and meaningful communal and structural investment to break its historic cycles. Based on our thematic analysis and literature review, four recommendations are offered: (1) Support and adopt policies to prevent or reduce structural violence; (2) Adopt multi-pronged strategies to transform dominant social norms associated with violence; (3) Establish standards and ensure adequate funding for violence prevention programs and services; and (4) Fund higher level ecological research on violence prevention and mitigation.

## Background

Violence is a critical public health problem associated with compromised health and social suffering that are preventable. It is also a complex phenomenon that involves a spectrum of behavioral and social interactions that vary across the lifespan and different social, political and economic contexts. The 2014 World Health Organization (WHO) Global Status Report on Violence Prevention indicates that the overall rate for victims of homicide worldwide in 2012 was 6.7 per 100,000 population [47]. While fatal violence is alarming to society and traumatic to the victims’ families and loved ones, it constitutes a small portion of interpersonal violence. Non-fatal physical, sexual and psychological violence make up the majority of interpersonal violence and these particularly affect children, women and seniors. For instance, an estimated 30 % of adult women worldwide experienced physical and/or sexual violence by an intimate partner (IPV) at some point in their lives [46], 22.6 % experienced physical abuse, and 36.3 % experienced emotional abuse as a child [33]. Data also show that 6 % of older adults reported significant abuse in the past month [8]. Furthermore, incidents of non-fatal violence are often under-reported, thus, actual rates are much higher.

In 2014, only few of the 133 countries surveyed by the WHO implemented prevention programs at a level commensurate with the scale and severity of the problem [47]. Canada fell short in elder abuse prevention, victim law reforms, and initiatives to promote gender equity [47]. The WHO [47] recommended that effective and sustainable global violence prevention efforts must be comprehensive and tackle a wide range of social conditions and structural determinants that fuel violence; that is, economic marginalization, ageism and gender inequality. The WHO’s recommendations imply that the notion of violence must be understood beyond interpersonal violence to make visible the impact of *structural violence*.

Structural violence, a term first coined by Johan Galtung [19], is defined as the difference between the potential and actual physical, mental, social and spiritual wellbeing of persons affected. Galtung asserted that interpersonal violence can only be understood in the context of structural violence, which is systemic in nature and often remains invisible. Expanding on Galtung’s work, Paul Farmer [17] illustrated that inequitable distribution of power and resources across different groups in society produces differential life chances that shape their everyday lived experiences. Wong [40] considers structural violence as a system of interlocking oppressions manifested in the form of social and economic deprivation, limiting marginalized people’s ability to reach their full physical, emotional, cultural and spiritual potential. She also emphasized that structural violence is an *avoidable* cause of health disparities that can be addressed through research, policy and practice.

The use of structural violence as an analytical lens to understand health disparities is aligned with evidence generated by research on social determinants of health in a neoliberal advanced capitalist globalized economy (see [4, 10]). As in other advanced capitalist countries, the 1970s marked a turning point in Canada from a post-war Keynesian Welfare state that focused on redistributive justice policies, to a free-market neoliberal state that emphasizes competitive individualism and consumption as a source of identity and means for social participation [9]. It is important to note that neoliberalism did not translate into total withdrawal of the state. Rather, neoliberalism has transformed state intervention from redistributive justice of social welfare for the poor to redistribution of wealth from the ordinary people to the elite; for example, in the form of *bailouts* or *corporate welfare* [1]. In Canada, neoliberal public policies and practices resulted in reduced access to welfare and the social security system. Examples include, welfare-to-work programs that required education and employment participation in order to receive benefits: deregulation of the market, privatization of public services, restructuring of the Unemployment Insurance program to what is now known as Employment Insurance: and the replacement of the Canadian Assistance Plan by the Canada Health and Social Transfer [3]. To a large extent, neoliberal practice perpetuates structural violence against vulnerable groups. For instance, neoliberal discourse of individual responsibility further marginalizes women who are welfare recipients when they are constructed as the cause of government fiscal deficits [6], or portrayed in the media and our popular imagination as lazy and undeserving freeloaders. Structural violence embedded in public policy and social institutions is invisible and yet powerful in perpetuating interpersonal violence, which is deemed to be private, random, and individual events. The interlocking cycle of structural and interpersonal violence disempowers individuals and communities, particularly those marginalized at the intersections of gender, race and class [5], and reinforces health disparities [16, 39].

Ryerson University’ Centre for Global Health and Health Equity, in dialogue with external research networks, organized and implemented a forum to explore issues relating to violence. The objectives of the Forum were to identify: (1) priority issues related to violence affecting different population groups in Canada, and (2) strategies to take action on priority issues to reduce violence-related health inequities in Canada. In this paper, we present findings from discussion and dialogue at the Forum, offer insights on the socio-political implications of these findings, and provide recommendations for action to reduce violence through research, policy and practice.

### Description of the forum and methods

The Global Health and Equity Forum was held in Toronto, Canada in 2014. Invitations were sent to hospitals, community health centres, non-profit community organizations working with vulnerable groups, universities, and public health units in the Greater Toronto Area. Over 60 researchers, health and social service agency staff, community advocates and graduate students attended the daylong Forum, which included presentations on structural violence, community violence, gender-based violence, and violence against marginalized groups. The presentations were followed by five concurrent roundtable discussions. Participants were invited to join one of five roundtables that interested them: children, youth, women, men and older adults. Each roundtable was attended by 10-12 participants; it was facilitated by a member of Centre for Global Health and Health Equity team and the discussion lasted approximately an hour. Being more participant-driven than researcher-driven, the Roundtable were considered to be an effective strategy for promoting critical dialogues. The composition of each Roundtable is presented in Table 1.

**Table 1:** Roundtable Participants

Type and No. of Participants	Children	Youth	Women	Men	Older Adults
Facilitator	1	1	1	1	1
Note-takers	1	1	2	1	1
Health/Social service providers	1	1	4	3	3
Community advocates	3	3	2	2	3
Undergraduate students	0	0	1	3	0
Graduate students	1	1	2	1	1
Other	4	4	4	4	1

Roundtable participants were asked to discuss priority issues related to violence based on their current professional experience and observations, and identify strategies for addressing these violence-related priorities and resulting health inequities. A graduate student or member of Centre for Global Health and Health Equity team was assigned to each roundtable as the note-taker to capture the composition of each roundtable group, and notes on the discussion. Notes from the roundtables were circulated to the facilitator of each roundtable for review and to insert additional reflective comments. Upon receiving all the reviewed notes, the first author used thematic analysis as the method to identify and organize findings into relevant themes and categories [34]. These themes were shared and discussed with the research team members who facilitated the roundtables to reach analytical consensus that inform this paper.

### Findings from the forum

Through thematic analysis, we identified four priority areas from the participants’ discussions:

1. structural violence perpetuates interpersonal violence
2. social norms of gender-based violence
3. violence prevention and mitigation programs; and
4. research gaps.
5. Structural violence perpetuates interpersonal violence
  The notion of structural violence was used by Forum participants to describe the historical, social and economic marginalization that contributes to interpersonal and community violence. Participants in many of the roundtable groups provided examples of factors related to structural violence, including poverty, gender inequity, transphobia, homophobia, racism, and other forms of discrimination and social exclusion. For instance, participants at the Women’s Roundtable identified the association between interpersonal violence and systemic discrimination. They suggested that underlying social inequities in the forms of women’s economic and legal dependency on men, economic and educational exclusion, and neoliberal practice which limits access and opportunities, all contribute indirectly to violence against women. As one participant explained, “People will deny that patriarchal values are still an issue today. We must shed light on the root causes; the sociological values that are embedded into our society and behavior (direct quote captured by note-taker).” Similarly, participants at the Men’s Roundtable highlighted men’s differential access to privilege and power when compared to women and children. However, participants also highlighted that in Canada and other White settler societies, not all men share similar access to privilege and power. Men of marginalized social positions experience structural violence in the form of neocolonialism, racism, homophobia, transphobia, ableism, economic marginalization and other intersecting oppressions that compromise their health and social wellbeing. At the Older Adult’s Roundtable, participants raised concerns about immigration policies, particularly with respect to sponsorship, which combine with racism, classism and ageism, to foster power imbalance and dependency in immigrant families. Participants at the Children and Youth Roundtable recommended that it is important for all people, but especially parents and families, to become aware of the impacts of structural violence on access to safe living environments, education, and aspiration for children and youth.
  An overarching theme that emerged across all roundtables was the importance of addressing violence at the level of public policy. Participants identified the need to target policy makers and government leaders to raise awareness of the impact of structural violence in the forms of policies and laws, and to push for commitment of resources to address the structural forms of violence (e.g., poverty, racism, sexism) because education alone is not sufficient to reduce violence-related inequities. Many participants identified the lack of intersectoral approaches (i.e., involving health, employment, social, educational, housing and criminal justice sectors) that require commitment and coordinated efforts across different levels of government and different ministries or departments. For example, effective strategies to reduce youth related violence must include increasing access to inclusive employment and educational opportunities for youth and providing financial and social supports for parents to help reduce the risks that are associated with violence and inequitable outcomes. Participants also suggested that decision-makers and administrators of institutions (e.g., hospitals, police, group homes, and the child welfare system) must examine how structural violence is produced and sustained through their organizational policies and practices that act as barriers to disempower individuals and communities. For example, racial profiling or the discriminatory practice by law enforcement officials to target individuals for suspicion of crime based on the individuals’ racialized background, ethnicity, religion or national origin is found to be common practice in Canada [7, 35].

2) Social norms of gender-based violence
  Participants across the different roundtables identified social norms as a powerful force behind interpersonal violence in different populations.
  For example, at the Women’s Roundtable, participants noted that in Canada and elsewhere, there exists a culture of gender-based violence manifested in a culture of rape and victim blaming that is very challenging to shift. Women continue to be objectified in the media, which produces and reproduces stereotypes, reinforces power differentials and normalizes violence against women (VAW). As one participant stated, “Women in advertisements become objects. They lose their face and humanism, which is the first step towards making violence more acceptable (direct quote recorded by note-taker).” Women felt that gender-based violence is embedded in Canadian social norms that do not value the equitable participation of women in leadership capacities.
  At the Men’s Roundtable, participants identified the need to engage men, especially marginalized men, to (re)define “what it means to be men”. They suggested that gendered norms often condone violence as an expected masculine role expectations and practice, which reinforce self-harm and violence against women and other men (e.g., violence in sports, dating violence, violence against gay men). Men who experience social and economic marginalization are often pushed into street economies that increased their involvement in violence.
  Participants at the Youth Roundtable highlighted that children and youth are also expected to fit with gendered norms and role expectation. In addition, they suggested that children and youth often encounter an additional layer of social norms based on age. Forum participants reported, based on their professional experience, that societal norms and adult judgments (e.g., from parents, teachers, and community workers) often produce negative stereotypes about children and youth that are stigmatizing and disempowering; children and youth in marginalized communities bear the brunt of these stereotypes.
  Many participants felt that shifting broader social norms and gendered expectations is a necessary prerequisite to the adoption of laws and policies to reduce structural violence and interpersonal violence. For example, social norms for gender-based interactions are taken up by children as they observe adults’ involvement and responses to violence. Furthermore, participants identified the need to deconstruct the neoliberal agenda and practice in which violence is constructed as individual behaviors, and freedom from violence is achieved through individual vigilance and efforts. They emphasized the need to make visible that interpersonal violence is produced through power relations and social structures, and the negative impact of intergenerational violence, as experienced by the Indigenous peoples of Canada and elsewhere. These forms of violence, especially among Indigenous populations, must be addressed through deliberate efforts based on the principles of social justice and equity.

3) Violence prevention and mitigation programs
  Forum participants noted that changing dominant social norms requires strategies of critical dialogue, stakeholder engagement, and political action at multiple levels: (1) at the societal level, there is a need to make visible how neoliberal practice, manifested in current regressive social welfare policies, reinforces structural violence that contributes to social and health inequities, including interpersonal violence; (2) at the community level, there is a need for sector leaders, service providers, activists and community members alike to recognize violence, in its myriad forms, as a priority for health and social wellbeing requiring collective action; and (3) at the individual and interpersonal level, there is a need for critical understanding of the (re)production of dominant social norms and gendered expectations that normalize violence behaviors.
  Participants also spoke about the advantage of engaging broader and more diverse stakeholder groups - including the media, faith leaders, health and social service providers, researchers, policy-makers, community members (including older adults, women, men, children and youth), advocates - as agents of change in transforming social norms that perpetuate violence. Although Forum participants recognized that most victims of intimate partner violence are women and most abusers are men, they considered it necessary to engage men in politicized popular education strategies that interrogate the practice of violence against women in the historical, social, economic and political contexts. They also emphasized the importance of promoting critical community dialogue about hegemonic masculinities and the consistent use of gender equity messages by leaders and influential figures.
  On a pragmatic note, Forum participants advocated for educational and skills development programs for the primary prevention of interpersonal violence. Examples provided by participants included school-based and community-based educational programming for children and youth that focus on gender relations and gender identities; family dynamics; healthy relationships; anger management, as well as supporting children and youth to develop skills in recognizing problematic behaviors associated with violence and abuse in the home environment, personal relationships, and beyond. Others identified training for health and social service providers as critical to improving consistency and comfort levels in the screening of individuals experiencing abuse and in providing inclusive and culturally safe care.
  Participants at the Women’s Roundtable recommended educating young women and men on VAW help address the general belief among younger generation that the issue of VAW has been resolved or is no longer relevant. They also suggested that educational programs do not focus only on VAW. As one participant shared, “You can teach the makeup of a respectful relationship and educate about conflict resolution without even having to mention violence”.
  Participants at the Men’s Roundtable recommended strategies and programming designed specifically for boys and men that focus on masculine identities, self-love, and mutually empowering relationships. They suggested the use of cross-sector partnerships that involve leaders and mentors from diverse sectors (e.g., arts, media, sports) to encourage boys and men in expressing their stress and negative emotions through non-threatening or non-harmful outlets such as martial arts and sports. They also emphasized the importance of providing safe gender-specific spaces for boys and men to critically deconstruct masculine expectations such as toughness, stoicism, and emotional disconnectedness as ways to promote their health and social wellbeing. At the same time, some participants identified mandatory community programs for male abusers as an important mitigating strategy to reduce VAW. It is recognized that the evidence to date to support this strategy is inconclusive.

4) Research gaps on violence
  Forum participants identified numerous research gaps related to violence. They felt that there is a need for comprehensive evidence derived from: systematic reviews on violence; community-based research that capture different forms of violence at the grassroots level; community-based interventions that inform violence prevention practice; and evaluation research on multi-sectoral services for individuals experiencing violence. Participants also highlighted the importance of implementation research, which provide evidence-informed strategies for effective program adoption and adaptation to reduce violence.
  There was consensus among participants on the need for research that identify and evaluate promising practices in violence prevention and mitigation. Furthermore, participants emphasized the need to translate this research evidence into products that are readily available and accessible to policy-makers and decision-makers in order to inform policy and practice. Some examples of comprehensive implementation evidences include: what works for different age-specific and gender-specific populations in different communities (e.g., neighbourhoods, ethno-specific, socio-economic); and how to create relevant and effective social marketing messages for different populations.
  Forum participants recommended the use of community-based and participatory action research to explore the experiences of violence in diverse populations and to capture perspectives of service providers and other community stakeholders. They were also aware of the challenges and barriers in establishing meaningful and sustainable community-research partnerships due to the historical level of community distrust and the potential competing interests among the stakeholder groups, particularly in the current context of neoliberal practice that promotes individualistic competitiveness, and the diminishing resources for non-profit organizations.

## Discussion

The priority issues for Canada raised by the Forum participants were consistent with current global scholarship and practice in violence prevention and mitigation. The recognition of interpersonal violence as a manifestation of structural violence is critical in shifting the focus of blame from marginalized communities that have been further victimized by neoliberal practice holds government and institutions accountable for the inequitable distribution of power and resources that produces and reproduces violence in the first place. This is especially true in Indigenous communities where (neo)colonialism, resulting in imposed loss of land, languages and cultures, and intergenerational trauma, has and continues to pose devastating impact on indigenous people’s individual and collective health and wellbeing, as evidenced in the high rates of poverty, unemployment, substance use, low educational attainment, family violence, disproportionate burden of physical and mental illnesses, and premature deaths (Health [2, 21, 23, 26]).

The second priority issue identified was consistent with existing evidences that demonstrate the strong association between dominant social norms and violent practices; for example, patriarchy and corporal punishment; gender inequities and normalized VAW; hegemonic masculinities and violence against gay men [24, 31, 37]. There is abundant empirical evidence to illustrate the perpetuating cycle of social norms and violence: values and beliefs that condone violence are shaped and reinforced by patriarchal ideologies; they, in turn, contribute to and perpetuate violent behaviours at the micro- (individual), meso- (community) and macro- (societal) levels and promote a greater tolerance of men’s violent behaviors [11, 28, 38]. Notions of male honor, male dominance, female subordination, female modesty and female chastity are exemplars of how patriarchal beliefs and fundamentalist religious doctrines intersect to perpetuate and sanction VAW [14, 20, 37]. Research evidence also suggests that children who witness violence or are victims of violence are more likely to adopt violent behaviors [27].

Most of the violence prevention and mitigation strategies identified by Forum participants are consistent with the evidence-based strategies recently endorsed by the WHO (2014) for the violence prevention and response efforts. These include: (1) developing safe, stable and nurturing relationships between children and their parents and caregivers [42]; (2) developing life skills and relationship skills in children and adolescents; for example, programs designed to help children and adolescents manage anger, resolve conflicts in a non-violent way and develop social problem-solving skills [43]; and comprehensive intimate partner violence (IPV) prevention interventions to reduce IPV perpetration and victimization among adolescents [12]; (3) promoting gender equality to prevent and reduce VAW [44]; (4) changing cultural and social norms that normalize and support violence [41]; and (5) victim identification, care and support programs [45].

One unique and important idea about violence prevention and mitigation proposed by the Forum participants was the engagement of intersectoral stakeholders that go beyond the conventional legal, health and social service sectors. Existing literature on intersectoral services for victims of violence tend to report on the effectiveness of the provision of legal, housing, financial and safety advice; and facilitation of access to and the use of community resources such as shelters, emergency housing, and psychological interventions [30]. Addressing the needs of victims with trauma-focused care, cognitive behavioral therapy or other low-intensity psychological interventions and mental health services can potentially mitigate the serious mental health outcomes of violence [15, 29]. However, our Forum participants emphasized the importance of engaging stakeholders and leaders beyond the conventional sectors to include stakeholders and leaders from faith-based, arts, sports and media sectors to transform dominant social norms and support boys and men to engage in positive and healthy outlets of stress and emotions. Their suggestion is critical in that it promotes critical dialogue about violence prevention in the social space of boys’ and men’s everyday life. The involvement of respected community leaders is considered to be very effective in changing social norms [13, 36].

Many international bodies, such as the WHO, International Confederation of Midwives, Pan-American Health Organization as well as provincial health agencies in Canada are increasingly looking at the role of healthcare providers in the provision of quality care defined in multidimensional terms such as respect, equity, access, patient centredness and effectivness. Quality also denotes care underpinned by respect for the human rights of women, children and marginalized groups (e.g., right to informed consent, right to refuse treatment, right to equal treatment, right to access healthcare, right to health, right to privacy, right to live) [18, 32]. In the context of violence prevention and mitigation, quality care denotes care providers’ competence in screening their clients for actual and potential experience of violence, and provision of care that maximize their clients’ safety and wellbeing and minimize the risk and impact of trauma associated with violence. Quality care also includes contributions to violence prevention through research, advocacy, policy and practice.

Finally, the research gaps identified by the participants are consistent with the current landscape of research on violence, which reflects an imbalance of research that focuses more on violence at the individual and interpersonal level (e.g., personal history of violence, interpersonal violence with peers, intimate partners or family members) and less on violence at the community level, whereby relationships are embedded in social spaces such as schools, workplaces, and neighborhoods, or structural violence at the societal level in the forms of social norms, institutional practices and public polices [25]. In 1999, the WHO Violence Prevention Alliance adopted an ecological framework developed by Heise [22] to illustrate how the complex interplay of individual, relationship, social, cultural and environmental factors are associated with intimate partner violence [25]. Research that promotes a better understanding of how socio-ecological factors interact to perpetuate or reduce violence is a key step to developing effective public health approaches to address violence.

## Limitations

While the organizing committee aimed to be inclusive in inviting individuals from diverse sectors, the Forum participants are not representative of all stakeholder groups. For example, there were fewer practitioners from law enforcement, education, and legal services, and fewer community members or public participants. Given the importance of community participation in, and community as the setting for violence prevention, the inclusion of the views of the public would have been informative and important. We relied on taking detailed notes to capture participants’ ideas and perspectives since audio-recording of these dialogues was not feasible in the context of a forum with many concurrent discussion taking place in the same room.

## Conclusion and Recommendations

The proceedings from the Global Health and Health Equity Forum underscore the importance of recognizing violence as a public health issue that requires immediate and meaningful communal and structural investment to break its historic cycles. In order to do so, it is important to bring together community members, grassroots organizations, researchers, policy-makers, educators, students and cross-sector stakeholders to advance discussions about effective policy and programming for different forms of violence. Given the nature of structural and interpersonal violence, any significant and lasting change will require intersectoral efforts. This Forum is just one example of the continued efforts needed to solidify violence prevention policies, services, and practices. However, we recognize that effective and sustainable solutions must surpass forums of discussion to actualize policies and regulations that can effectively transform oppressive social structures, address the gaps in violence prevention and mitigation, and reinstate societal responsibility for violence prevention.

Based on our thematic analysis of the information from the forum, the members of the Centre for Global Health and Equity at Ryerson offers the following recommendations:

1. Support and adopt policies to prevent and reduce structural violence.
  Reducing social disparities through social policy reform is essential for both violence prevention and reduction. Social policy reforms must address the root cause of violence, that is, inequitable distribution of power and resources resulting in differential access to social and economic opportunities between the dominant groups and the marginalized groups. While these broad considerations must be central to social policies in general, understanding the specific needs of marginalized groups negatively impacted by racism, sexism, ageism, homophobia, transphobia, poverty and other oppressions is a priority. In recognition of violence as a structural issue, finding solutions requires a political and intersectoral approach (i.e., involving health, employment, social service, educational, housing and criminal justice sectors). For example, strategies to reduce youth related violence should include increasing employment and educational opportunities for youth and providing financial and social supports for parents to help reduce the risks that are associated with violence and inequitable outcomes. Similar actions are needed to inform and ensure that institutions (e.g., hospitals, police, group homes, and the child welfare system) review and revise their organizational policies and procedures to address structural violence that creates barriers to providing inclusive and respectful services to marginalized individuals and communities.
2. Adopt multi-pronged strategies to change dominant social norms associated with violence.
  Since a multiplicity of external and internal pressures function to maintain dominant cultural and social norms, multi-pronged approaches that engage diverse stakeholders to influence community opinions, inform mass media messages and transform public policy or legislation must be used to address violence. Mass media campaigns can be used to convey messages about empowering relationships and social interactions to broad populations via television, radio, the Internet, newspapers, magazines and other social mediums. Legislation is an important strategy in changing cultural perceptions and social norms when violent behaviors are redefined as a criminal offence and the offenders have to face legal consequences. Political will and social accountability are also critical because the presence of legislation without the necessary infrastructure of public education, access to legal services, and person-centred services for victims of violence does not guarantee that the legislation will be carried out effectively.
3. Establish standards and ensure adequate funding for violence prevention program and services.
  Comprehensive research evidence is needed to establish promising practices and reorient services to meet the individual and collective needs of affected groups and communities to reduce violence and its related impact. Community-based services must be strengthened through sustained government funding to promote community empowerment in the continuum of violence prevention and reduction programs. Adequate funding for coordinated and sustainable capacity building programming at the community level is critical to the establishment of effective responses to interpersonal and community violence and to reduce the loss of community capacity to address violence due to resource competition among stakeholder groups.
4. Fund higher-level ecological research on violence prevention.
  The current research agenda, with its focus on violence at the personal or interpersonal level does not provide the necessary knowledge and evidence to inform policy development and change. Adequate resources are needed to support research that investigate the outcomes of upstream policies on violent prevention, the impact of intersectoral interventions to transform dominant social norms, and the effectiveness of population-specific interventions on primary violence prevention.
